# Proton pump inhibitor use and risk of pneumonia: a self-controlled case series study

**DOI:** 10.1007/s00535-023-02007-5

**Published:** 2023-06-14

**Authors:** John Maret-Ouda, Joni Panula, Giola Santoni, Shaohua Xie, Jesper Lagergren

**Affiliations:** 1 Department of Medical Epidemiology and Biostatistics, Karolinska Institutet, Stockholm, Sweden; 2 Centre for Clinical Research Sörmland, Uppsala University, Eskilstuna, Sweden; 3 Upper Gastrointestinal Surgery, Department of Molecular Medicine and Surgery, Karolinska Institutet and Karolinska University Hospital, Stockholm, Sweden; 4 School of Cancer and Pharmaceutical Sciences, King’s College London, London, UK

**Keywords:** Pulmonary infection, PPI, Population-based, Confounding

## Abstract

**Background:**

Recent research indicates that use of proton pump inhibitors (PPIs) is associated with pneumonia, but existing evidence is inconclusive because of methodological issues. This study aimed to answer whether PPI-use increases risk of pneumonia while taking the methodological concerns of previous research into account.

**Methods:**

This population-based and nationwide Swedish study conducted in 2005–2019 used a self-controlled case series design. Data came from national registries for medications, diagnoses, and mortality. Conditional fixed-effect Poisson regression provided incidence rate ratios (IRR) with 95% confidence intervals (CI) for pneumonia comparing PPI-exposed periods with unexposed periods in the same individuals, thus controlling for confounding. Analyses were stratified by PPI-treatment duration, sex, age, and smoking-related diseases. Use of histamine type-2 receptor antagonists (used for the same indications as PPIs) and risk of pneumonia was analysed for assessing the validity and specificity of the results for PPI-therapy and pneumonia.

**Results:**

Among 519,152 patients with at least one pneumonia episode during the study period, 307,709 periods of PPI-treatment occurred. PPI-use was followed by an overall 73% increased risk of pneumonia (IRR 1.73, 95% CI 1.71–1.75). The IRRs were increased across strata of PPI-treatment duration, sex, age, and smoking-related disease status. No such strong association was found between histamine type-2 receptor antagonist use and risk of pneumonia (IRR 1.08, 95% CI 1.02–1.14).

**Conclusions:**

PPI-use seems to be associated with an increased risk of pneumonia. This finding highlights a need for caution in using PPIs in individuals with a history of pneumonia.

**Supplementary Information:**

The online version contains supplementary material available at 10.1007/s00535-023-02007-5.

## Introduction

Proton pump inhibitors (PPIs) are among the most commonly prescribed medications globally. They are mainly used for the treatment or prevention of gastric acid-related upper gastrointestinal tract disorders, including gastroesophageal reflux disease and ulcer [1]. PPI-use has increased over the last decades and there seems to be a degree of over-prescription of PPIs both regarding treatment duration and indications [2–4]. Serious adverse outcomes may have an impact not only on individual patients, but also on public health and healthcare.

Recent evidence suggests that PPI-use increases the risk of community-acquired pneumonia (from here on called pneumonia) [5]. A proposed mechanism is that the decreased gastric acidity following PPI-treatment alters the gut and oral microbiome, and micro-aspiration of such a microbiome might cause pneumonia [6, 7]. In addition, proton pumps have been identified in the respiratory tract, and PPI-use may alter the microbiome in the respiratory tract, possibly inducing pneumonia [8]. Meta-analyses of observational studies have indicated that PPI-treatment increases the risk of pneumonia [9, 10], while a recent randomized controlled trial (RCT) found no such association [11]. The associations found in the meta-analyses might be biased by confounding and selection, and the null finding of the RCT could be explained by low statistical power and strict patient selection [5, 12]. Our recent review of the existing evidence concluded that the evidence of an association between PPI-use and pneumonia is inconclusive, mainly because of methodological concerns [5]. This finding prompted us to perform the present study.

With the aim to clarify whether and, if so, to which extent PPI-use increases the risk of pneumonia, and avoid the above-mentioned methodological issues hampering the existing literature, we conducted a large study of an unselected population where confounding was controlled for by letting the participants be their own controls.

## Methods

### Design

This was a population-based and nationwide Swedish study during the study period from July 1, 2005 to December 31, 2019. We used a self-controlled case series design, which is an attractive alternative to more conventional study designs in its ability to control for time-invariant confounders [13]. This design means that the number of events during exposed periods is compared to the number of events during unexposed periods for the same individual. Because participants act as their own controls, confounding by known, residual, and unknown factors is counteracted. This design is suitable only for studies examining transient exposures, e.g. PPI-use, in relation to outcomes with short induction time and duration, e.g. pneumonia [13]. The study was approved by the Regional Ethical Review Board in Stockholm.

### Cohort

The source cohort was the Swedish Prescribed Drugs and Health Cohort (SPREDH), which has been presented in detail elsewhere [14]. In brief, this is a population-based Swedish nationwide cohort, created for research examining how prescribed medications influence the risk of various outcomes. SPREDH includes all Swedish residents who dispensed at least one of the most commonly prescribed medications, including PPIs, during the study period, i.e., 9,091,193 individuals. These represent the vast majority of the adult Swedish population. SPREDH consists of merged individual-level data from four national and well-maintained Swedish health data registries:

*1. The Prescribed Drug Registry* includes data on all medications prescribed and dispensed in outpatient care in Sweden [15, 16]. The recording started July 1, 2005 and is highly accurate and almost 100% complete because all dispensations are automatically issued from the pharmacies to this registry. The medications are classified according to the Anatomical Therapeutic Chemical classification (ATC) system.

*2. The Patient Registry* contains data on all Swedish inpatient care from 1987 onwards, and all specialist outpatient care since 2001 [17]. The registry holds data on all diagnoses and surgical or medical procedures according to the International Classification of Diseases (ICD) codes with nearly 100% completeness [17]. Information regarding dates of admission and discharge, patient age and sex, and healthcare providers is also available.

*3. The Cancer Registry* holds data on all malignant tumours diagnosed in Sweden from 1958 onwards with 96–98% coverage [18].

*4. The Cause of Death Registry* records all deaths of Swedish residents with 100% completeness and accuracy regarding the date of death [19].

All individuals living in Sweden have a unique personal identity number, which is used in all registries included in the study, making it possible to link the registry data for each participant [20].

The present study included all adult individuals (aged 18 years or above) in the source cohort (SPREDH) with a pneumonia diagnosis recorded after cohort entry (index date). Patients entered the study at the index date if the age was ≥ 18 years or one year after the last pneumonia episode before the index date, whichever was the latest. Patients were followed up until end of the study period or death, whichever occurred first. All data in the study came from Swedish national registries with high completeness, hence, there were no known missing data.

### Exposure

The study exposure was PPI-treatment (ATC-code A02BC). The PPI-exposure period was counted from the date of dispensation of any PPI until 30 days after ending the treatment (the latter to ensure restitution of gastrointestinal microbiome after ceased PPI-treatment). The duration of treatment was estimated from the defined daily dose, i.e. the average daily dose for the PPI [21]. The time of PPI-exposure was divided into three categories: first month of treatment (1–30 days), second and third month of treatment (31–90 days), and more than three months (> 90 days) of treatment. Because PPIs can be prescribed during pneumonia hospitalizations and be continued after discharge [3], we excluded a period of 60 days before the dispensation date of PPI-treatment from the unexposed period and accounted for separately as pre-exposure period. Outside any PPI-treatment period or pre-exposed period, the participants were considered unexposed.

Treatment with a histamine type-2 receptor antagonist (H2RA; ATC-code A02BA) was included as a separate exposure only for assessing the validity of the findings for PPI-use. H2RAs are used for similar indications as PPIs, and there is no known mechanism linking H2RA-use with pneumonia.

### Outcome

The outcome was bacterial or viral pneumonia (ICD version 10, codes J10.0, J11.0, J12-J16, J17.0-J17.1, or J18). Both primary and secondary pneumonias were included. Patients were censored from the study for 90 days after the date of a pneumonia diagnosis to ensure full recovery before a possible next episode of pneumonia was counted [22].

### Statistical analysis

Incidence rate ratios (IRR) with 95% confidence intervals (CI) of pneumonias during PPI-exposed person-time compared to during unexposed person-time were calculated using a conditional fixed-effect Poisson regression model. Except for the inherent control of confounding by the self-controlled series design, the IRRs were also adjusted for age (categorized into 18–49, 50–69, or ≥ 70 years) and calendar period (year 2005–2010, 2010–2014, or 2015–2019). Analyses were stratified by age (18–49, 50–69, and ≥ 70 years), sex (men and women), and smoking-related diseases (yes and no; ICD version 9, codes 491, 492, and 496; ICD version 10, codes J40-J44 and J47). Sensitivity analyses dismissed the 90-day censoring after a previous pneumonia episode that was used in the main analyses. All analyses followed a pre-planned protocol and were conducted by an experienced biostatistician (GS) using the statistical software STATA version 15MP (StataCorp, College Station, TX, USA).

## Results

### Participants

The study included 519,152 participants with at least one pneumonia episode. Among these, the total number of pneumonias was 677,086 (one patient could have more than one episode of pneumonia). The mean follow-up was 12.3 years (interquartile range 9.2–13.3 years). The study compared 307,709 periods of PPI-exposure with 507,016 periods without PPI-use. The distribution of sex, age, and calendar period was similar during PPI-exposed and unexposed periods (Table 1).

**Table 1:** Characteristics of 519,152 participants by use of a proton pump inhibitors (PPI)

Exposure	No PPI*	PPI*
Number of periods	507,016 (62.2%)	307,709 (37.8%)
Person-years	3,780,049 (80.0%)	943,143 (20.0%)
Sex		
Male	256,625 (50.6%)	149,752 (48.7%)
Female	250,391 (49.4%)	157,957 (51.3%)
Age (interquartile range), years		
< 50	89,324 (14.0%)	38,565 (10.8%)
50–69	205,805 (32.3%)	109,133 (30.7%)
≥ 70	341,861 (53.7%)	208,142 (58.5%)
Calendar year (interquartile range)		
2005–2010	468,549 (39.8%)	180,741 (33.0%)
2010–2014	411,869 (35.0%)	203,777 (37.3%)
2015–2019	297,334 (25.2%)	162,509 (29.7%)
Smoking-related diseases	91,375 (16.1%)	65,459 (19.4%)
Number of pneumonias, mean (standard deviation)		
	0.94 (0.76)	0.64 (0.86)

^*^Patients can contribute to both unexposed and exposed periods multiple times and in time-variant parameters (age, calendar year, smoking-related diseases)

### Proton pump inhibitors

The overall risk of pneumonia during PPI-exposed periods was increased by 73% compared to non-exposed periods (adjusted IRR 1.73, 95% CI 1.71–1.75) (Table 2). The adjusted IRRs of pneumonia were increased for each of the duration periods of PPI-use compared to unexposed periods (IRR 2.59 [95% CI 2.55–2.63] for 1–30 days of treatment; IRR 2.25, [95% CI 2.22–2.28] for 31–90 days of treatment; and IRR 2.20 [95% CI 2.17–2.23] for > 90 days of treatment) (Table 2). Stratified analyses revealed increased risks of pneumonia across age groups, sexes, and smoking-related diseases status, although the risk estimates were more pronounced among patients of older age, male sex, and without smoking-related diseases (Table 3). The sensitivity analysis without censoring for 90 days after pneumonia showed similar results as in the main analyses with censoring (Supplementary Table 1).

**Table 2:** Incidence rate ratio (IRR) with 95% confidence interval (CI) of pneumonia comparing proton pump inhibitor (PPI) treatment periods with unexposed periods

Exposure	Pneumonias (number)	Crude IRR (95% CI)	Adjusted IRR (95% CI) *
No PPI	479,103	1.00 (reference)	1.00 (reference)
PPI	197,983	2.44 (2.42–2.47)	1.73 (1.71–1.75)
No PPI			
Baseline	437,599	1.00 (reference)	1.00 (reference)
60 days prior to PPI	41,504	4.25 (4.20–4.31)	3.64 (3.59–3.68)
PPI			
First month (1–30 days)	18,809	3.01 (2.96–3.06)	2.59 (2.55–2.63)
Second and third month (31–90 days)	27,570	2.69 (2.65–2.73)	2.25 (2.22–2.28)
After the third month (> 90 days)	151,604	3.50 (3.46–3.54)	2.20 (2.17–2.23)

^*^Adjusted for age and calendar year

**Table 3:** Incidence rate ratios (IRR) with 95% confidence interval (CI) of pneumonia comparing proton pump inhibitor (PPI) treatment periods with unexposed periods in stratified analyses

Characteristic and exposure	Pneumonias (number)	Crude IRR (95% CI)	Adjusted IRR (95% CI) *
Age < 50 years			
No PPI	69,032	1.00 (reference)	1.00 (reference)
PPI	11,444	1.79 (1.74–1.85)	1.40 (1.35–1.45)
Age 50–69 years			
No PPI	114,682	1.00 (reference)	1.00 (reference)
PPI	48,621	2.27 (2.23–2.31)	1.71 (1.68–1.75)
Age ≥ 70 years			
No PPI	295,389	1.00 (reference)	1.00 (reference)
PPI	137,918	2.38 (2.35–2.41)	1.79 (1.76–1.81)
Men			
No PPI	248,869	1.00 (reference)	1.00 (reference)
PPI	98,608	2.64 (2.60–2.67)	1.88 (1.85–1.91)
Women			
No PPI	230,234	1.00 (reference)	1.00 (reference)
PPI	99,375	2.27 (2.23–2.30)	1.60 (1.57–1.62)
No smoking-related diseases			
No PPI	394,843	1.00 (reference)	1.00 (reference)
PPI	146,101	2.47 (2.44–2.46)	1.84 (1.82–1.86)
Smoking-related diseases			
No PPI	84,260	1.00 (reference)	1.00 (reference)
PPI	51,882	1.51(1.48–1.54)	1.23 (1.20–1.25)

^*^Adjusted for age and calendar year

### Histamine type-2 receptor antagonists

The risk of pneumonia during H2RA-exposed periods was at the most only marginally increased compared to unexposed periods (adjusted IRR 1.08, 95% CI 1.02–1.14) (Supplementary Table 2).

## Discussion

This study indicates that PPI-use is followed by a substantially increased risk of pneumonia. This association was consistent across PPI-exposure durations, age groups, sexes, and smoking-related diseases status. H2RA-treatment was not associated with any clearly increased risk of pneumonia.

Some methodological issues of the study require a discussion. Among strengths is the self-controlled design, which is ideal for examining PPI-exposure in relation to pneumonia outcome because of the short exposure time and the short outcome induction time [13]. This design made it possible to control for all types of confounding, including unknown confounders. The population-based design with very high participation rate counteracted selection bias enabled generalizability to other similar populations. The complete follow-up of all participants by using their personal identity numbers linked to complete national registers excluded losses to follow-up [20]. The nationwide setting provided good statistical power. The almost total lack of association between H2RA-use and pneumonia supports the absence of confounding by indication and that the association between PPI-use and pneumonia is specific.

Among weaknesses is potential influence of time-varying confounders, e.g. other medication (e.g. steroids) as well as protopathic bias, which can occur if PPI-use is influenced by subclinical stages of a pneumonia. However, the consistency of the results across duration periods of PPI argues against such errors. Another limitation is that the lack of data on discontinuation of the PPI-treatment despite having dispensed the prescription. However, the discontinuation rate should be low considering the low prevalence of side-effects among short-term users of PPI. The fact that the short-term duration of PPI-use showed a strong association with pneumonia further argue against this being an issue. No data were available on over-the-counter use of PPI, but PPIs are available only in small packages at a much higher cost in Sweden, making it likely that most patients get prescriptions. Moreover, any discontinuation and over-the-counter use of PPIs should occur at random and therefore lead to dilution of the association rather than explaining it. There was no information regarding the indication for the PPI-treatment. Eradication treatment of *Helicobacter pylori* includes antibiotics, which may also be used in the treatment of pneumonia, but PPIs used for eradication of *Helicobacter pylori* in Sweden is mainly (84.9%) prescribed as a single combination prescription (ATC-code A02BD), which was not included as PPI-exposure in this study [23]. Additionally, antibiotic treatment of *Helicobacter pylori* would decrease risk estimates of pneumonia during PPI-treatment, not the opposite. Because PPI-use is not an established risk factor for pneumonia, the occurrence of pneumonia should not affect subsequent PPI-exposure. The death rate associated with pneumonia is low, and any bias due to a shortened follow-up should thus be limited. The sensitivity in assessing pneumonia using ICD-codes in the Swedish Patient Registry is not known, but might have been low. However, such misclassification of the outcome should not be dependent on the PPI-exposure and thus not explain the association identified, but rather dilute them. Thus, the true association might be stronger. The increased risk of pneumonia found during a pre-exposed period for up to 60 days has been found also in other studies, and might be explained by underlying increased risk of pneumonia in patients preceding a PPI prescription.

Whether PPI-use increases the risk of pneumonia is a matter of much interest and debate. Two meta-analyses have found results in line with those of the present study. In a meta-analysis of 26 studies and a total of 226,769 patients with pneumonia, PPI-use was associated with a 49% increased risk of pneumonia (odds ratio 1.49, 95% CI 1.16–1.92) [9]. A smaller, but more recent meta-analysis of 7 studies, with 65,590 cases of pneumonia, found a 66% increased risk of pneumonia (odds ratio 1.66, 95% CI 1.22–2.25) [10]. However, both these meta-analyses were based mainly on case–control studies, with an inherent risk of confounding and selection bias, which makes the findings uncertain. Confounding by indication for PPI-use is a particular concern because gastroesophageal reflux disease, a main indication for PPI-therapy, may increase the risk of pneumonia [12, 24, 25]. In a recent and large cohort study from Canada, the United Kingdom, and the United States, 4,238,504 new users of NSAIDs using PPI for ulcer prevention were assessed in an attempt to avoid the influence of gastroesophageal reflux disease, showing no association between PPI-use and pneumonia (odds ratio 1.05, 95% CI 0.89–1.25) [24]. Another recent study of 48,451 patients in the United Kingdom applied a self-controlled design and found a slightly increased risk of pneumonia during PPI-use (IRR 1.19, 95% CI 1.14–1.25) [25]. An RCT of 17,598 patients with stable cardiovascular disease who initiated treatment with aspirin and/or rivaroxaban and were randomized to use PPI (pantoprazole) or a placebo found no increased risk of pneumonia among the PPI-users compared to the placebo group (odds ratio 1.02, 95% CI 0.87–1.19) [11]. However, among concerns with this RCT are the low statistical power for assessing pneumonia and the short follow-up [26]. A recent review (from our group) of the available literature on the topic concluded that the role of PPI-use in the aetiology of pneumonia remains unanswered [5]. The present study adds support for PPI-use as a risk factor for pneumonia.

A mechanism by which PPI-use might lead to pneumonia is by altered gastrointestinal and oral microbiomes [6, 7]. PPIs can cause gastric and oral overgrowth of *Streptococcus* [27], and there is evidence for bacterial exchange between gastric and lung fluids [27]. Thus, it is proposed that the altered microbiomes might colonize the respiratory tract, predisposing to pneumonia. Gastric and duodenal bacterial overgrowth is clearly more common in PPI-users than in H2RA-users, which can explain the major difference between these drugs regarding the risk of pneumonia [28]. Another mechanism could be mediated by proton pumps in the respiratory tract, whereby PPIs may alter the microbiome of the respiratory tract locally and induce pneumonia [8].

The results of this study stress the relevance of using PPIs for the correct indications and avoid overuse. PPIs are the cornerstone of the treatment for acid-related gastrointestinal tract disorders, but the prevalence of PPI-use increases every year, which does not correspond to the incidence of acid-related diseases [2, 5]. A review of administrative data from the United States found that 61–73% of patients on PPI-therapy did not have a valid indication for its use [3]. The increased risk of pneumonia during PPI-treatment could be detrimental in some patients because pneumonia can be a serious disease, sometimes lethal [29].

In conclusion, this large and population-based study, which counteracted confounding utilizing a self-control design and validated the specificity by also examining H2RA-use, indicates that PPI-use strongly increases the risk of community-acquired pneumonia. These findings highlight the importance of carefully considering the indication and duration of PPI-treatment.

## Supplementary Information

Below is the link to the electronic supplementary material.

Supplementary file1 (DOCX 22 KB)
