# Supplementary material for: Proton pump inhibitor use and risk of pneumonia: a self-controlled case series study
Source: J Gastroenterol. 2023 Jun 14;58(8):734–40. doi: 10.1007/s00535-023-02007-5 (PMC10366235; doi:10.1007/s00535-023-02007-5)
Supplement: Supplementary file 1 — Supplementary file1 (DOCX 22 KB) [file 535_2023_2007_MOESM1_ESM.docx]

**SUPPLEMENTARY MATERIAL**

**Supplementary Table 1. Sensitivity analysis: Incidence rate ratios (IRR) with 95% confidence interval (CI) of pneumonia comparing proton pump inhibitor (PPI) treatment periods with unexposed periods without censoring for 90 days after pneumonia.**

| **Exposure** |  | **Pneumonias**  **(number)** | **Crude IRR**  **(95% CI)** | **Adjusted IRR (95% CI) *** |
| --- | --- | --- | --- | --- |
| **No PPI** |  | 605,006 | 1.00 (reference) | 1.00 (reference) |
| **PPI** |  | 261,972 | 2.38 (2.36-2.40) | 1.69 (1.67-1.71) |
|  |  |  |  |  |
| **No PPI** | **Start** | 550,195 | 1.00 (reference) | 1.00 (reference) |
|  | **60 days prior to PPI** | 54,811 | 4.20 (4.15-4.26) | 3.57 (3.52-3.62) |
| **Duration of PPI** | **<31 days** | 25,417 | 2.97 (2.93-3.02) | 2.53 (2.49-2.57) |
|  | **31-90 days** | 37,248 | 2.65 (2.61-2.69) | 2.19 (2.16-2.23) |
|  | **>90 days** | 199,307 | 3.41 (3.37-3.45) | 2.14 (2.12-2.17) |

*Adjusted for age and calendar year.

**Supplementary Table 2. Incidence rate ratio (IRR) with 95% confidence interval (CI) of pneumonia comparing incidence during current histamine-2 receptor antagonist (H2RA) treatment with unexposed periods.**

| **Exposure** |  | **Pneumonias**  **(number)** | **Crude IRR**  **(95% CI)** | **Adjusted IRR (95% CI) *** |
| --- | --- | --- | --- | --- |
| **No H2RA** |  | 475,717 | 1.00 (reference) | 1.00 (reference) |
| **H2RA** |  | 3,386 | 0.94 (0.89-1.00) | 1.08 (1.02-1.14) |
|  |  |  |  |  |
| **No H2RA** | **Baseline** | 474,677 | 1.00 (reference) | 1.00 (reference) |
|  | **60 days prior to H2RA** | 1,040 | 1.12 (1.04-1.20) | 1.35 (1.25-1.45) |
| **Duration of H2RA** | **<31 days** | 773 | 0.93 (0.86-1.01) | 1.13 (1.04-1.24) |
|  | **31-90 days** | 979 | 0.89 (0.82-0.96) | 1.08 (1.00-1.17) |
|  | **>90 days** | 1,634 | 1.08 (1.00-1.18) | 1.19 (1.10-1.30) |

*Adjusted for age and calendar year in groups.
